# Pain in Persons with Disorders of Consciousness

**DOI:** 10.3390/brainsci12030300

**Published:** 2022-02-23

**Authors:** Nathan D. Zasler, Rita Formisano, Marta Aloisi

**Affiliations:** 1 Concussion Care Centre of Virginia, LTD, Henrico, VA 23233, USA; 2 Department of Physical Medicine and Rehabilitation, Virginia Commonwealth University, Richmond, VA 23298, USA; 3 IRCCS, Santa Lucia Foundation, 00179 Rome, Italy; r.formisano@hsantalucia.it (R.F.); m.aloisi@hsantalucia.it (M.A.)

**Keywords:** disorders of consciousness, pain, suffering, severe brain injury, ethics, pathophysiology, assessment, management

## Abstract

Pain and suffering in persons with disorders of consciousness (DoC) remain poorly understood, frequently unaddressed or inadequately addressed, and controversial on numerous levels. This narrative literature review will address a number of critical issues germane to pain and suffering in this challenging group of patients, providing an introductory overview of the topic, perspectives on current knowledge regarding pain pathoanatomy and pathophysiology, and a review of common pain generators and factors that can lead to the chronifcation of pain. Caveats on bedside pain assessment challenges, as well as electrophysiologic and neuroimaging findings in these patients, will also be explored. Pain management techniques, including non-pharmacological and pharmacological, will be reviewed. Ethical considerations in the context of pain and suffering in persons with disorders of consciousness will round out the review prior to our concluding comments.

## 1. Introduction

In the practice of medicine, pain is often encountered consequential to disease and/or acquired brain injuries. A recent revision of the definition of pain has been proposed by the International Association for the Study of Pain (IASP) [1], in which pain has been defined as “some unpleasant sensory and emotional experience associated with actual or potential tissue damage or described in terms of such damage.” Furthermore, pain is always a subjective experience, influenced to varying degrees by biological, psychological, and social factors.

Pain can present in many different ways, in part dependent upon the underlying peripheral versus central mechanisms of the same, as well as the acuity versus chronicity of the pain condition. Acute pain is generally treated very differently from chronic pain due to differences in pathoetiology, as well as the frequent secondary consequences of chronic pain, such as anxiety and depression. Chronic pain is typically defined as pain lasting for longer than 6 months, although the timeframe is empirical in nature and others have advocated for 3 months or more, while some pain practitioners have opined that chronic pain is any pain that lasts beyond the timeframe for the normal resolution of the original pain generator [2]. Chronic pain syndromes occur when the pain disorder impacts quality of life as far as mood, sleep, fatigue, libido, and participation in vocational and avocational activities. Pain can persist following the acute and subacute periods, even when the pathologic condition causing the pain has resolved. Pain chronicity can be due to both peripheral and central sensitization mechanisms and/or the non-resolution of the pain generator, such as that which might occur with a chronic physical condition/impairment.

Pain is an inherent part of the human experience. Pain disorders may cause impairment in human function as well as disability-related consequences. When pain is experienced as an unpleasant physical sensation or emotion, it is typically expressed through the conscious experience of the same or externalized in the form of verbalizations, vocalizations, and/or behavioral responses, such as grimacing, crying, or agitation. In neurologically impaired patients, pain, whether consciously perceived or not, may be accompanied by increased spasticity as well as autonomic activation (i.e., hypertension, tachycardia, tachypnea, sweating, pupillary changes, etc.), including the worsening of dysautonomic symptoms [3,4,5,6].

Pain and suffering are distinct entities. Pain is the physiologic manifestation of nociception (which refers to the detection of noxious stimuli by nociceptors), although pain itself may not always be linked to a stimulus and does not necessarily correlate with the degree of injury severity [7]. Nociception, or the neural process of encoding noxious stimuli, refers to the perception (conscious or not) of such stimuli [8], eliciting the activation of an extensive cortical network. Non-nociceptive pain does not require a noxious stimulus as that which might occur in allodynia or neuropathy [9]. Additionally, pain is not necessarily a direct consequence of nociception and involves the interaction of multiple inputs [10]. Pain mediation requires a complex multidimensional neuromatrix that integrates pain sensations with complex functions involving affect, memory, and autonomic self-regulation [11,12,13,14]. Suffering entails an individual’s emotional reaction to the perception of pain and may involve an array of different responses, including frustration, denial, indignation, and self-indignation, among other responses. The relationship between pain and suffering is complex, as pain is not necessarily always associated with suffering, and suffering is certainly not always associated with pain, whether physical or emotional. Most suffering is induced by negative emotions or situations, and endorsing that a given patient is suffering would implicitly require the clinician to have evidence that such behavioral responses were in fact occurring. There remains debate regarding the boundaries of pain and suffering that the practitioner should be cognizant of in clinical practice [9]. As clinicians, avoiding or modulating both pain and suffering should be one of the primary goals for our patients, regardless of their clinical condition.

Other factors must be considered when assessing pain, including sex differences in pain perception and cultural influences on the subjective experience of pain. In general, women tend to be at increased risk for chronic pain and may experience more severe pain. Women have greater pain sensitivity, reduced pain inhibition, and enhanced pain facilitation compared to men; a factor to be considered in future pain studies. There are also studies that suggest differential responses between the sexes to pharmacotherapeutic treatment for pain [15] that require further study in healthy controls with findings extending accordingly to other patient populations. Cultural influences have also been shown to play a role in a person’s subjective pain experience, as well as how they manage their pain experience [16,17].

In the context of treating persons with a disorder of consciousness (DoC), including vegetative state (VS)/unresponsive wakefulness syndrome (UWS) and minimally conscious state (MCS), one of the more challenging areas of practice is that of assessing and managing pain [18,19,20,21,22]. The number of individuals with DoC has increased due in part to progress in intensive and acute neurosurgical critical care, bringing increased attention to this group of challenging patients, regardless of the etiology of their DoC. One consequence of this progress is a trend toward increased awareness of the limitations of dogmatic early prognostication and a shift to delaying decisions about withholding and/or withdrawing care in these patients early after severe brain injury [23]. As noted by Schnakers and Zasler [18], these factors have driven greater levels of introspection regarding the clinical assessment and treatment of persons with DoC in general, and more specifically regarding issues of pain assessment and management. These advances in DoC knowledge and practice have also brought to light medicolegal as well as ethical considerations in the context of ongoing controversies regarding pain and suffering in persons with DoC, leading to a multitude of clinical management challenges extending well beyond the boundaries of end–of–life decisions [24,25,26].

This narrative literature review was carried out using PubMed, Google Scholar, Scopus, and Web of Science to scour the literature for relevant studies on pain and DoC. The review was conducted using the following key words related to the topics at hand: pain, suffering, severe brain injury, ethics, pathophysiology, pain assessment, and pain management. As part of the review, we also surveyed references used in previously published articles on the topic to identify any other relevant papers that may have been missed in the literature review.

## 2. Pain Pathoanatomy and Pathophysiology

**Figure 1:**
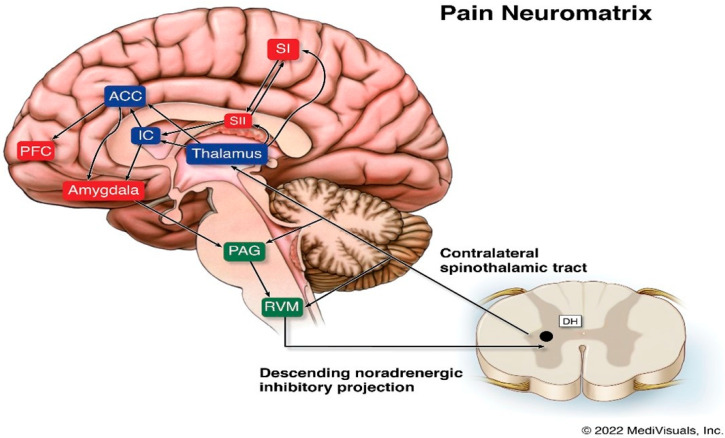
**Legend:** Schematic diagram of the pain neuromatrix involving cortical and subcortical structures. ACC, anterior cingulate cortex; DH, dorsal horn; IC, insular cortex; PAG, periaqueductal gray; PFC, prefrontal cortex; RVM, rostral ventral medulla; SI, primary somatosensory cortex; SII, secondary somatosensory cortex.

The pain neuromatrix in the intact CNS is complex and still not fully understood [27] (see Figure 1). The pathophysiology of pain following brain insult or injury is more challenging, as the assessment of the exact underlying, potentially multisite, pathoanatomy of the injury is not always known and, for the reasons noted above, the post-injury neuropathology tends to not be of major help in determining a person’s potential for perceiving pain, whether nociceptive or non-nociceptive.

The ascending spinothalamic tract is a major pain pathway that carries nociceptive, thermal, and non-discriminative touch information to the thalamus. These axons then connect to the posterior limb of the internal capsule, and then to the postcentral gyrus and posterior paracentral lobule of the parietal lobe, where they mediate sharp pain sensations. Poorly localized pain sensations associated with the emotional features of pain are mediated via the intralaminar nuclei and end in the insular and rostral cingulate gyrus. The primary and secondary somatosensory, insular, anterior cingulate, and prefrontal cortices are the areas involved in qualitative aspects of pain, localization, intensity, and duration [28,29,30].

Suffering and the cognitive evaluative aspects of pain are mediated by the insular, anterior cingulate, and prefrontal cortices as well as subcortical limbic structures [31,32]. Spatiotemporal brain activity reorganization has been found to accompany the transition to chronic pain, with pain representation gradually shifting from sensory to emotional and limbic structures. Pain-induced limbic gamma oscillations have interestingly been shown to be related to pain perception in persons with DoC [33,34]. There is preliminary evidence that some patients in UWS may be able to perceive the affective components of pain through limbic circuit activation [33], which should give clinicians at least some pause to being dogmatic that those in a UWS cannot perceive pain.

Descending pathways transmit information from various regions of the brain, including the cortex, hypothalamus, and amygdala, to the periaqueductal gray in the midbrain, which further project to the somatosensory cortex. Spinothalamic tract damage, including to the thalamic nuclei, may be involved with persistent post-TBI pain. Dopaminergic system dysfunction due to alterations in dopamine signaling has been shown to occur after TBI and may be involved with persistent pain complaints. Hypothalamic dopamine projections to the spinal cord may augment, rather than suppress, pain, indicating that the effects of dopamine on pain are not necessarily consistent. Various other pathophysiological processes, including, but not necessarily limited to, neuroinflammation, neurodegeneration, and axonal damage have been implicated in various animal and human models of post-TBI pain, as have synaptic changes and genetic mechanisms [35,36].

Dysfunctional descending inhibition can also contribute to pain conditions resulting from brain injury. The descending system is of particular importance in the regulation of pain arising from injured peripheral tissues, such as the nerves, osseous structures, skin, and muscles. Descending inhibition is modulated by norepinephrine from the locus coeruleus and serotonin from the rostroentromedial medulla, and targets the spinal cord dorsal horn and the trigeminal nucleus. Alterations in norepinephrine neurotransmission and consequential motor and descending nociceptive inhibition have been found to play essential roles in pain modulation. Based on current knowledge, which includes neuroimaging studies, chronic pain may occur secondary to the disruption of descending pathways affecting nociceptive signaling mechanisms [35].

## 3. Pain Generators in Persons with DoC

The main causes of pain during early recovery post-trauma are fractures (both axial and extremity), solid organ injuries, soft tissue injuries, and inserted tubes (i.e., tracheal, nasogastric, urinary catheters, etc.). Invasive procedures, such as surgical interventions, central line insertions, and intravenous lines can all be pain generators [22,37]. In the subacute setting, similar issues are responsible for pain and may continue to serve as pain generators. Clinicians should be alert to clinical changes (vital signs, pain behaviors, including agitation, diaphoresis, and increased tone) on attempts at weaning pain medication. If pain generators persist, these reflexive responses to pain (which in and of themselves can be physiologically stressful to the patient’s system) should be addressed through appropriate pharmacological and non-pharmacological prescription. In select patients with a DoC, withdrawal of pain medications can lead to a conscious pain experience and potentially suffering.

Transition to lower levels of care does not imply that there is no longer a need for due diligence with regard to pain surveillance and treatment. As patients medically stabilize and the primary issue is not “life or death,” there tends to be greater attention paid to assessing pain if there is evidence of pain responsivity. Following acute care, common causes of pain may include spasticity, rigidity, dystonia, contractures, musculoskeletal pain, complex regional pain syndrome, shoulder subluxation, scoliosis, thalamic (central) pain, and/or skin breakdown [38,39]. Clinicians should always think about therapy interventions, such as range of motion exercises, as potentially painful experiences for individuals with a DoC [40], which has clinical implications as to whether we should be pharmacologically prophylaxing for pain preemptory to such procedures.

Evidence strongly supports the mechanisms of central sensitization as being contributory to pain evolving to a chronic state [41]. Various neural areas have been found to be associated with both structural and functional changes (including in corticolimbic networks) as related to pain chronification [42,43]. Such mechanisms are generally not found to contribute to acute and subacute pain. There are now a number of different approaches utilized to treat chronic pain conditions that are theorized to be due to central sensitization phenomena, including potentially neuromodulatory techniques [39]. There are also a number of conditions that occur in persons with DoC that are unrelated to central sensitization, but are common causes of pain, including intracranial pressure changes, immobility, contractures, spasticity, skin breakdown, and urinary tract infections (with dysuria), among other conditions. Obviously, each of these conditions can be treated through appropriate surgical and/or non-surgical interventions that must be within the treatment armamentarium of any physician involved with the management of persons with DoC. Please refer to Table 1 for a list of common pain generators in persons with DoC.

## 4. Pain Assessment in Persons with DoC

Historically, accurate bedside assessment and the determination of consciousness of patients with a DoC has a high error rate [44,45,46]. Misinterpretation of behavioral signs may lead to errors that have ethical and clinical, as well as clinicolegal, implications relative to decisions regarding prognosis, treatment, and end–of–life decision making. In the assessment of non-communicative patients, it is essential to discriminate between reflex and higher-order behavioral responses [30]. Bedside examination of patients with DoC may determine their awareness/consciousness based on indirect clinical indicators, such as mimic reactions, crying, shouting, groaning, and grimacing. Newer evidence suggests that there may be additional clinical indicators of consciousness in the post-acute period, including the rate of spontaneous eye blinking [47].

Several scales have been validated to assess the presence of nociception in patients unable to communicate, such as newborns, infants, adolescents [48,49,50], and older and demented persons [51] (see Table 2). Since self-report pain evaluations, such as the Visual Analogic Scale (VAS) [52], are not possible to administer to non-communicative patients, the aforementioned scales are based on specific behavioral observations, such as noxious stimulus localization, restlessness or agitation, body movements, facial expressions, emotional reactions (grimaces, crying, screaming, and shouting), verbalizations, vocalizations, verbal complaints, and other non-verbal pain indicators [53]. Specific recommendations have been proposed by an American Task Force for all patients not able to communicate verbally (non-verbal patients) to receive appropriate pain management interventions [54].

The variability of the pain sources in persons with prolonged DoC (PDoC) may lead to different individual reactions to the same noxious stimulation (pressure on the fingernail bed); thus, individualized pain stimulation in such patients should be used [54]. However, assessing nociception and behavioral responses to pain stimulation in individuals with severe brain injury and disorders of consciousness (DoC) still represents a challenge to any clinician charged with caring for such patients [18].

Several behavioral scales have been authored in an attempt to allow a more objective assessment of pain in this challenging population [54]. The Nociception Coma Scale (NCS) [55,56,57] is a tool that was designed to assist in the objective assessment of pain in persons with a DoC. The measure relies on the observation of the motor response (non-flaccid, abnormal posturing, flexion withdrawal, and localization), verbal response (non-verbalization, groaning, vocalization, and intelligible verbalization), visual response (none, startle response, eyes movement, and fixation), and facial expression (non-oral reflexive/startle response, grimace, and crying), following a defined and standardized noxious stimulation (i.e., pressure on the fingernail bed using an algometer). The visual item was subsequently removed from the NCS, and a revised version of the scale, the Nociception Coma Scale–Revised (NCS–R), was proposed by Chatelle [56] as it was found that the exclusion of the visual item did not change the overall assessment findings.

Sattin et al. [58] found lower pain pressure thresholds in participants with DoC compared to healthy controls. Pathologic pain responses, such as allodynia, have the potential to alter responses to stimuli, even though they may not be considered as painful. Personalized stimuli (e.g., hand opening, upper limb abduction, and head mobilization) have been proposed for pain stimuli response assessment on the NCS and NCS–R due to the alterations in pain pathway functions that might affect the response to standard pressure on the fingernail bed. Indeed, a preliminary study revealed that personalized, tailored painful stimulation was able to elicit more behavioral responses than standard stimuli [59]. An adapted version of the NCS–R using personalized stimulation (PS) may produce more intentional and specific responses to pain-inducing maneuvers. There is a clear and apparent need to conduct further research examining individual thresholds and the nature of the pain stimulus applied in patients with a DoC.

An integrated patient-centered approach for the assessment of physical pain in which clinical measures and bedside behavioral observations are assessed may improve treatment and rehabilitation outcomes. Furthermore, investigating pain perception in such patients through the use of a personalized source of nociception and pain may avoid non-specific, unreliable, and potentially harmful noxious assessments (as with standard pain scales) [22,60] and may provide tools for revealing nociception, even in the absence of any response to the standard clinical bedside exam. Through the use of data generated from the most involved caregivers (nurses, physiotherapists, physicians, and relatives), along with clinician-performed qualitative analysis, may provide more reliable data to assess for otherwise occult pain perception and potentially suffering in those who cannot otherwise convey their discomfort [54,59].

The Brain Injury Nociception Assessment Measure (BINAM), a specific measure of nociception, was developed for non-communicative patients with severe TBI. The BINAM consists of ten behavioral and physiological items and has been found to be reliable and feasible to administer, with accurate scores obtainable in about 10 min [61]. One of the advantages of BINAM in comparison with NCS–R is that the use of the latter scale may be less sensitive in the assessment of pain in patients with VS/UWS due to their low functional level [62]. Moreover, the subscores in the motor and verbal response categories of the NCS–R may be problematic to score in patients who are intubated, trached, paretic, and/or aphasic [63,64]. The effects of activity and analgesia with acetaminophen on BINAM have also been evaluated, demonstrating that the resulting score is largely independent of the level of consciousness or agitation [61,62,63,64].

Pain perception in persons with DoC remains a controversial issue, particularly due to the fact that there is evolving evidence of the presence of residual pain experience in some patients in VS/UWS [65,66,67,68,69,70]. Managing and treating pain may improve a patient’s ability to participate in assessments as well as therapies, reduce psychomotor agitation, improve sleep, and potentially even global outcomes. In this vein, the Italian Ministry of Health passed a law [71] that patients must receive proper pain treatment, and that pain assessment must be included in the patient’s evaluation. Bagnato et al. [72] found that admission NCS–R and CRS scores paralleled the levels of consciousness, but that, unlike the CRS–R, the NCS–R scoring was not related to the 6-month outcomes in UWS patients. The prior finding is seemingly somewhat paradoxical, given that prior studies showed similar correlations between higher NCS–R scores and CRS scores and the historical correlation of early higher CRS scores with better longer-term outcomes.

Proper assessment and treatment of pain in patients with DoC who may experience pain at a conscious level and potentially even suffer, yet be unable to demonstrate to the outside world their experience, should be a goal of all practitioners working with this challenging group of patients. A patient-centered and multidisciplinary approach is advocated to facilitate the assessment of pain in patients with a DoC to improve the diagnostic accuracy and ultimately the quality of management of a given patient’s pain experience [59]. It should also be noted that there is currently no consensus on which pain assessment scale is preferred in persons with a DoC based on either consensus or blinded prospective controlled studies, although there is a trend toward providing “personalized” stimulation.

## 5. Electrophysiological Assessment

Different painful stimuli have been proposed to reveal EEG reactivity to pain in patients in comas [60]. Ronga et al. [73] studied event-related potentials (ERPs) elicited by transient nociceptive stimuli in normal subjects. They found that nociceptive ERPs do not simply detect the novelty of the painful stimulus, but instead are mainly determined by their saliency, which consists of an increase in nociceptive stimulation that is more salient than a decrease in stimulation intensity. Based on these findings, further studies should evaluate differential responses based on pain stimulus intensity and saliency.

In a recent scoping review [74], the neurophysiological underpinning of pain in prolonged DoC (PDoC) was examined. Pain perception assessment was noted to potentially assist in reducing the misdiagnosis rate in this patient population. The data indicate that patients with UWS have more dysfunction in the pain neuromatrix connectivity areas compared to individuals in MCS, emphasizing that pain perception may be more likely as the overall level of consciousness improves. There are noteworthy exceptions, since some patients with a UWS show pain-related cortical activations that partially overlap with the activations observed in individuals in an MCS. This suggests that some patients with UWS may have residual brain functional connectivity supporting the somatosensory, affective, and cognitive aspects of pain processing that would facilitate covert cognition assessment and thus reduce misdiagnosis [75,76,77]. Further studies examining the utility of electrophysiological evaluation are certainly warranted to assess their role in pain assessment in DoC, as well as predictive utility in signaling emergence.

De Salvo and colleagues [78] studied whether laser-evoked potentials (LEPs) could objectively demonstrate the residual pain perception capacity in patients in VS/UWS and MCS. The authors performed a cross-sectional observational study focusing on the role of LEP examination to confirm its use as a neurophysiological marker of pain perception. The study group consisted of 13 VS and 10 MCS patients with inter-group LEP analysis showing significant differences in post-anoxic N2P2 latency, amplitude, and a trend in N2P2 latency in brain trauma. Interestingly, correlation analysis showed a significant relationship between N2P2 amplitude and CRS–R scoring only in the post-traumatic VS/UWS group. These latter findings lead to the detection of potential markers of conscious pain perception in patients with a DoC and have potentially important implications for clinical management.

In a study by De Tommaso and colleagues, five patients with a UWS and four patients with an MCS, as well as eleven age- and sex-matched controls, were examined [66]. Auditory, visual, non-noxious electrical, and noxious laser stimulation were used during evoked potential data collection. The laser-evoked potentials (LEPs) were recognizable in all cases. Only one MCS patient showed a reliable cortical response to all the employed stimulus modalities. Significant N2 and P2 latency prolongation occurred in both VS and MCS patients. The presence of a reliable cortical response to auditory, visual, and electric stimuli was able to correctly classify patients with UWS and MCS with 90% accuracy. The laser P2 and N2 amplitudes were not correlated with the CRS–R and NCS–R scores, while the auditory and electric-related potential amplitudes were associated with the motor response to pain and consciousness recovery. The investigators concluded that LEPs did not seem to correlate to responsiveness per se, but to motor responses to painful stimuli. Other neurophysiological studies utilizing event-related evoked potentials (ER–EP) have shown that the activation of the N400 wave after noxious stimulation was unable to differentiate between patients with VS/UWS and MCS (“near VS”) with any clarity [79].

## 6. Neuroimaging

The two main Positron Emission Tomography (PET) studies on pain perception in individuals with UWS demonstrated contradictory results. Laureys and colleagues demonstrated that painful electrical stimulation of the medial nerve in 15 subjects with UWS due to various etiologies was able to activate only primary cerebral areas for pain perception, such as the brainstem, thalamus, and primary somatosensory cortex [68]. Kassubek and colleagues [67], using similar methodology, studied seven persons with long term anoxic UWS and found that activation of somatosensory cortical areas was associated with conscious pain perception. The difference between the two studies might be due to the time post-brain injury that the PET imaging was performed, as the Laureys patient group was much more acute than the patient group studied by Kassubek.

Other PET studies have demonstrated a global disconnection between the secondary and primary cerebral areas for pain perception [80,81]. Boly et al. [82] confirmed a reduction of the activation of all of the areas of the “pain cerebral matrix” in individuals with UWS, without precise differentiation between subjects. Comparable results of the activation of primary, but not secondary, cerebral areas in response to external stimulation have been obtained by neuroimaging techniques, such as functional Magnetic Resonance (fMRI) techniques [65,69].

The activation of only primary cortical areas for pain perception might be interpreted as a lack or reduction of conscious pain perception in persons with UWS due to the failed activation of the secondary “pain matrix” (cingulate cortex, insular, and frontoparietal network). These types of studies are critical to pursue to improve our clinical abilities to assess for covert cognition, as well as pain perception and suffering, given the high percentage of misdiagnosis between UWS and MCS in nearly half to one-third of chronic cases of DoC [44,83,84,85,86,87].

In partial conflict with the reported role of the cingulate cortex in conscious pain perception is the observation that cingulotomy may alleviate the emotional–affective component of pain, yet pain perception remains [88]. Similarly, the neuropathological results from the brain of Karen Ann Quinlan at autopsy after surviving 10 years in a UWS demonstrated that the most severe damage was in the thalamus, whereas only minimal to moderate neuronal loss was found in the insular, cingulate area, and orbito-frontal cortex. It was reported, nonetheless, that the patient withdrew all four limbs upon noxious stimulation with a needle point [89], as occurs in most patients in UWS. Based on those observations, Klein [90] concluded that at least some individuals with VS, especially those with preserved anterior cingulate cortex and thalamic function, might have some level of conscious pain perception. Other reports favoring subcortical pain perception [91] are supported by the experience of infants with anencephaly, in whom reactions to pain similar to healthy subjects are preserved [18,92,93].

## 7. Pain Management

The management of pain in persons with DoC has not been studied in any structured manner, and much of clinical practice in this regard is drawn from the general pain management literature. Currently, there are no available guidelines (evidence or consensus-based) on pain management in this population. There remains substantive debate regarding issues pertaining to differentiating reflexive responses to pain from cortically mediated pain perception [20,94]. The issue of the assessment and detection of suffering consequential to pain, the ability of persons with a DoC to suffer (even if at a decreased level from controls who were not brain injured), and the treatment of same have received little to no formal research attention.

During acute care, practitioners will be limited to some extent in terms of the array of medications that might be reasonable to use to treat pain in this patient population due to the issues of neurological and neurosurgical instability. Any drug prescription that adversely impacts neurologic presentation should obviously be avoided as much as possible or alternatively used with extreme caution (drugs with sedative properties, proconvulsants, and agents that may impede cognition and/or neurological recovery, such as anticholinergics and dopamine antagonists, respectively, among other medications). Additionally, consideration should be given to medications with reversible effects (e.g., opiate reversal with naltrexone) whenever there is the question of medication effect versus the deterioration of neurological status [39].

For neurologically compromised patients with response limitations, such as those that would be seen in persons with DoC, prophylactic pain management should be practiced based upon injuries sustained and clinical presentation. This should include both pharmacological and non-pharmacological interventions (see Table 3). Critically ill patients with a DoC, like other neurocritically ill patients, pose unique challenges for pain assessment as well as management. In this setting, pharmacologic prophylaxis of pain should be considered in all persons with a DoC, given the difficulty in the assessment of pain in this group of patients and the challenges associated with bedside assessment of pain [18,95]. As noted by Whyte [96], “The cost of analgesic or anesthetic treatments to patients in VS(UWS) who also have disorders or are undergoing procedures that are known to produce pain is small compared with the risk of failing to treat a patient who, at the time of such a procedure, might be able to experience pain subjectively.”

Clinicians need to be cognizant of the negative potential clinical impacts of pain (even in patients in a UWS) as related to “reflexive” responses to nociceptive stimuli, including increased tone/posturing, elevated intracranial pressure, masking of neurological signs of progress, and potential adverse neuroendocrine responses [37]. From a non-pharmacological standpoint, appropriate splinting, tone-reducing positioning, regular turning, routine bowel and bladder programs, general hygiene, appropriate and timely nail care, and close skin monitoring can be helpful in preventing, as well as modulating, pain [39,97]. Neuromodulation may also play a role in modulating certain types of pain, including migrainous headaches and neuropathic pain. Numerous techniques now exist to administer such treatments, including, but not limited to, deep brain and motor cortex stimulation, peripheral nerve stimulation, and non-invasive treatments, such as repetitive transcranial magnetic stimulation, transcranial direct current stimulation, and transcutaneous electrical nerve stimulation including supra-orbital, supratrochlear, and occipital. The role that behavioral techniques, such as relaxation therapy, meditation, or music therapy [98], may have in pain modulation in someone with a DoC has yet to be explored.

A patient with a DoC suffering from chronic pain should be treated just as aggressively as a patient with acute or subacute pain. Even in chronic cases, there are times when aggressively addressing pain generators may facilitate neurorecovery, as has been seen with intrathecal baclofen therapy. It has been theorized that the reduction of spasticity and pain in patients with a DoC is a key factor that may impact appropriate diagnosis, as well as positively influence recovery [94,99,100,101]. It should be remembered that some of the commonly used pharmacological agents used for the treatment of brain injury-related conditions may also have concurrent pain modulation properties, such as antiseizure, antispasticity, GABAergic, and antidepressant drugs. Indeed, central pain sources have been reported in patients with DoC. Spasticity was found in 89% of a sample of patients with DoC and was positively correlated with pain scores measured by means of Nociception Coma Scale–Revised (NCS–R); antispasticity medications were found to have the potential to diminish pain responses in such cases [38]. Neuroperipheral pain sources may also be present, such as in Critical Illness Polyneuropathy, Critical Myoneuropathy, and ICU-Acquired Weakness [102]. Drugs that are GABAergic, such as gabapentin and/or pregabalin, may be useful for pain management in the aforementioned disorders.

When discontinuing/weaning medications that may have never been prescribed for pain management, but may have analgesic properties, it is critical to monitor the patient for withdrawal symptoms (as may be appropriate depending on the medication) and, more importantly, evidence of breakthrough pain. Other drugs commonly used in persons with a DoC, such as beta-blockers (propranolol) for dysautonomic symptoms and NSAIDs for periarticular neurogenic heterotopic ossification, may also help modulate the subjective pain experience [103].

The approach to pharmacological management should be hierarchical relative to starting with the lowest-risk agent with potentially the greatest therapeutic benefit for decreasing pain. For mild pain, aspirin, acetaminophen, and NSAIDs should be first-line agents. For moderate pain, high-dose aspirin or acetaminophen, oral NSAIDs, newer-generation NSAIDs, such as cyclooxygenase II inhibitors (now limited to the single agent, celecoxib in the USA), injectable NSAIDs (such as ketorolac), mixed opiate analgesics with aspirin or acetaminophen (with or without caffeine), and tramadol can be considered. For pain generators that are associated with severe pain, the medications to consider would include parenteral opiates (morphine sulfate being the standard agent), mixed agonists/antagonists (pentazocine, nalbuphine), and partial agonist opiates (buprenorphine). Other agents, such as antidepressants, antiseizure medications, and atypical agents, such as memantine, can also be considered. Stimulants, such as methylphenidate, can be prescribed on an adjunctive basis with opioid analgesics to help manage opioid-induced sedation and cognitive impairment, but may be contraindicated in the early post-injury period [39]. Some patients may present with both opioid-sensitive and opioid-insensitive pain at different sites and due to different etiologies. NSAIDs, tricyclic antidepressants, antidepressants, such as venlafaxine or duloxetine, tizanidine, antiepileptic medications, and various topical medications (some of which may need to be produced through compounding pharmacies as these may be available), among other pharmacotherapeutic options, may be considered for opiate-insensitive pain [39].

Palliative sedation and pain control remain controversial, particularly as related to any determinations of withholding or withdrawing care [104]. There is no international consensus for practice regarding the ethical and legal aspects of the withdrawal and withholding of care. The thresholds for palliative sedation and what drugs to use for the same or their dosages remain debated and without international guidelines. In such situations, the primary goal should be to make the patient as comfortable as possible in terms of limiting pain and suffering [26,94].

## 8. Ethical Considerations

The ethical debates regarding the clinical and clinicolegal aspects of the assessment and treatment of persons with a DoC have not evolved in parallel with our advances in clinical assessment and treatment. There are numerous ethical consternations that arise in this context, including how we go about assessing pain in patients who may not be able to externally manifest responses to the same and the implications of causing a given patient pain and potentially making them suffer. There are also ethical dilemmas that we, as clinicians, must face when we see a patient who appears to be in pain and suffering consequently, but we cannot determine with a degree of medical probability whether this is in fact the case. Similarly, we should not be clinically erratically comfortable that the patient is not experiencing pain or suffering when there is an absence of any clinical indicators of the same based on bedside assessment or, for that matter, based upon some neurodiagnostic assessment. Another area of significant debate and controversy pertains to the withdrawal of hydration and nutrition in persons with a DOC in the context of end–of–life care. As an example of the aforementioned, Ms. Terri Schiavo’s end–of–life care did not include the use of prophylactic pain medications, such as opiates [105]. More recently, specific investigations with the inclusion of caregivers in the bedside behavioral assessment of persons with prolonged DoC (PDoC) [106] and the use of advanced diagnostic techniques have been proposed, at least during artificial nutrition and hydration (ANH) withdrawal, to address ethical and other concerns associated with potential pain and suffering during such interventions [26].

Literature on the incidence and prevalence of chronic central pain after severe traumatic brain injury (TBI) [107,108] suggests that we could not exclude hyperpathia in patients with DoC due to trauma. Indeed, hyperpathia and neurogenic pain may be responsible for some of the clinical features of psychomotor agitation observed in the recovery phases from DoC [109,110].

Future research on differentiating pain perception from suffering in patients with DoC is mandatory, not only to protect persons who are not able to express their discomfort, but to reassure caregivers of our ethical commitment toward their loved ones and the family [111].

## 9. Conclusions

As noted by Fins and Bernat [110], all clinicians should advocate for routine universal pain precautions in the context of neuropalliative care given the potential for the presence of covert consciousness [75,76,77]. Given the multidimensional nature of pain and suffering in the context of working with non-communicative patients and their families, it is essential that continued efforts be dedicated to scientific research in this critical area of brain injury medicine.

It is of common clinical experience that persons with DoC may express mimic reactions of sufferance, such as crying, shouting, and screaming, due to mobilization or from other sources of possible pain, such as visceral pain (constipation, bladder infections, etc.).

Therefore, the prophylactic use of pain medications has been recommended in all non-communicative patients, not only in patients in an MCS, but also in those in a UWS [18].

Families and caregivers are often worried about the possibility that their loved one is in pain and/or suffering, and yet we, as clinicians, cannot provide definitive answers in response to these concerns. Much has been accomplished in translational and experimental research that is applicable to the care and neurorehabilitation of patients with a DoC following severe acquired brain injury, but the major existential and emotional weight remains on the relatives and caregivers. Future research should focus on the use of advanced diagnostic techniques to investigate the “internal machinations” of non-communicative patients, methodologies to reduce misdiagnosis as related to missing the presence of covert consciousness, as well as covert pain perception and suffering, the use of new technologies, such as brain–computer interfaces and eye-gaze communication systems, to improve functional communication, and methods to improve the quality of life of patients with DoC and their families.

In summary, to better provide informed, innovative, and ethical care to those patients with DoC who are non-communicative and potentially experiencing pain and/or suffering in any phase of care, we must improve our scientific knowledge regarding pain and suffering assessment and management. In this context we cannot ignore or in any way minimize the role of family and/or caregivers, and must make better efforts to provide evidence-based and timely education, as well as supportive counseling regarding the phenomenon of pain, as well as its assessment and treatment in this group of patients. Future studies examining neurodiagnostic techniques to better define and explore a patient’s pain experience will be critical, as will studies assessing pharmacotherapeutic as well as non-pharmacological methods for pain management with an ultimate goal of the development of clinical practice guidelines for pain assessment and management in this special, yet challenging, patient population [112]. Multimodal assessments that include an appropriately focused bedside neurological and neurobehavioral examination, as well as neurophysiological testing, should be advocated for to enhance our ability to opine on a patient’s pain perception capacities, improve the differential diagnosis of the specific DoC, and guide patient-specific management as well as family education [74].

## Figures and Tables

**Table 1:** Common Pain Generators in Persons with DoC.

Central/thalamic pain
Complex regional pain syndrome
Constipation
Dystonias
Indwelling devices
Infectious processes—pneumonia, urinary tract infections
Invasive procedures
Low or high intracranial pressure
Myofascial pain
Neuralgic pain
Neuropathic pain
Neurogenic heterotopic ossification
Neuromusculoskeletal scoliosis
Post-fracture pain
Range of motion attempts
Shoulder subluxation
Skin breakdown/pressure sores
Soft tissue contractures
Soft tissue injuries
Solid organ injuries
Spasticity, rigidity, dystonia

**Table 2:** Pain Assessment Tools in Non-Communicative Patients.

Scale	Patient Group
Children and Infants Post-operative Pain Scale (CHIPPS)	Newborns, Infants, and Adolescents
Face, legs, activity, cry and consolability (FLACC)	Newborns, Infants, and Adolescents
Pain Assessment in Advanced Dementia (PAINAD)	Geriatric/Dementia
Nociception Coma Scale (NCS)	DoC
Nociception Coma Scale–Revised (NCS–R)	DoC
Nociception Coma Scale–Revised–Personalized Stimulation (NCS–R–PS)	DoC
Brain Injury Nociception Assessment Measure (BINAM)	Severe Traumatic Brain Injury

**Table 3:** Pain Management Caveats in Persons with a DoC.

Understand the likely pain generators
Approach to treatment in a hierarchical fashion
Start with non-pharmacological interventions first in cases of suspected mild to amimimoderate pain and assess response
When pain is suspected to be severe, use both pharmacologic and non-pharmacologic interventions to optimize pain modulation
When prescribing medications, start low and go slow
Monitor for side effects
Continue to assess pain via use of specialized DoC pain measures and/or neurodiagnostic strategies as available
Always make attempts to wean pain medications, unless there is a clinical indication for chronic administration

## Data Availability

Not applicable.
